# Aldehydes, Aldehyde Metabolism, and the ALDH2 Consortium

**DOI:** 10.3390/biom12060763

**Published:** 2022-05-30

**Authors:** Freeborn Rwere, Xuan Yu, Che-Hong Chen, Eric R. Gross

**Affiliations:** 1 Department of Anesthesiology, Perioperative and Pain Medicine, School of Medicine, Stanford University, Stanford, CA 94305, USA; frwere@stanford.edu (F.R.); xyu2018@stanford.edu (X.Y.); 2 Department of Chemical and Systems Biology, School of Medicine, Stanford University, Stanford, CA 94305, USA

## 1. Introduction

The discovery of aldehydes dates back to 1774 when Carl Wilhelm Scheele synthesized acetaldehyde [1]. However, its structure was not completely understood until Justus von Liebig in 1835 determined its constitution and named “aldehyde” to its chemical group (R-CHO), which was derived from the Latin phrase “alcohol dehydrogenatus” (meaning alcohol without hydrogen) [2]. Following their discovery, aldehydes triggered the growth of modern perfumery [3], while also revolutionizing the agriculture and manufacturing industries.

In addition to the exposure of aldehydes from these environmental sources, cells also endogenously produce aldehydes as signaling molecules. Aldehydes are highly reactive within the cell, and can form adducts leading to DNA damage and alter protein activity and/or function [4]. Understanding the impact that reactive aldehydes have on the cell is of great importance in humans, as these aldehyde-induced adducts can trigger pathophysiology leading to cancer, diabetes, cardiovascular, and neurodegenerative diseases [5].

Intracellular aldehydes can be neutralized by aldehyde-metabolizing enzymes, including those from the aldehyde dehydrogenase superfamily (ALDH). In particular, the importance of aldehyde dehydrogenase 2 (ALDH2) in aldehyde metabolism has come to the forefront of precision health, more so in the past decade. This is because an inactivating ALDH2 genetic variant is present for nearly 8% of the world population that limits aldehyde metabolism. With aldehyde exposure, the inactivating ALDH2 variant leads to greater risks of cancer and cardiovascular disease. In efforts to gather researchers across the world involved in aldehyde research and aldehyde metabolism, we sent out a request for this Special Issue in *Biomolecules*. This call for papers is conducted in in parallel to other international efforts to bring together a community with interests in aldehyde and aldehyde research. 

For this editorial, we highlight the research regarding aldehydes within this Special Issue in *Biomolecules*. In addition, we also describe the ALDH2 STAR consortium in order to raise awareness regarding how genetics influence aldehyde toxicity and aldehyde metabolism. 

## 2. Highlights of the *Biomolecules* Special Issue

**Figure 1:**
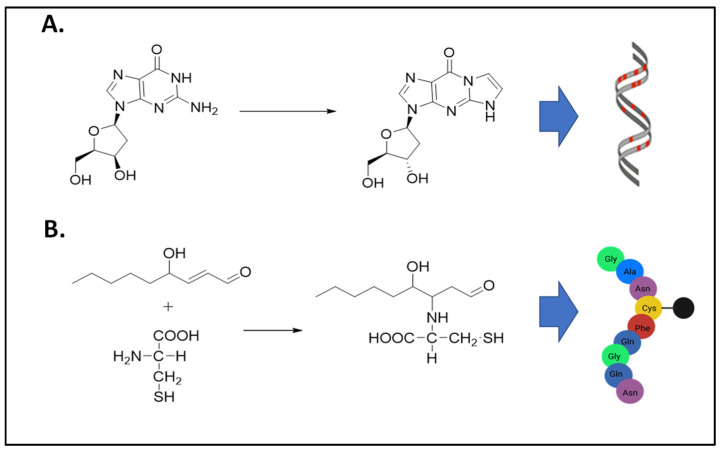
Exogenous and endogenous aldehydes cause DNA and protein adduct formations. (**A**) Formation of *N*^2^-etheno-2′-deoxyguanosine DNA adduct from deoxyguanosine and α,β-unsaturated aldehydes as products of lipid peroxidation. (**B**) Formation of a 4-hydroxynonenal (4-HNE) protein adduct from the reaction between 4-HNE and a cysteine residue. In addition to cysteine adducts, 4-HNE protein adducts can also form on histidine or lysine amino acids.

For this Special Issue, the nine original articles and two review articles on aldehyde toxicity and metabolism represent research from laboratories that span across four continents and include seven countries. Aldehydes are toxic to the cell as they can cause DNA damage and protein adduct formation (Figure 1). As discussed below, the body of work for this Special Issue identifies the continued need to examine aldehyde toxicity and metabolism directly caused by alcohol consumption in addition to aldehydes generated from sources other than alcohol.

### 2.1. Aldehyde Exposure from Alcohol

When consuming alcohol, the key enzyme responsible for converting acetaldehyde to acetic acid is aldehyde dehydrogenase 2 (ALDH2). Furthermore, ~560 million people worldwide are intolerant to alcohol due to an inactivating genetic variant in aldehyde dehydrogenase 2 (*rs671*), known as ALDH2*2 [5,6]. For heterozygotes (ALDH2*1/*2), this leads to a 40–60% reduction in ALDH2 enzymatic activity [7,8,9]. How long ago did this genetic variant appear in humans? To address this question, Lin et al. identified that the ALDH2*2 genetic variant originated ~7935 years ago (6000 B.C.), using the long-haplotype analysis of residents from Keelung, Taiwan, and Guangzhou, China [10]. Furthermore, when examining 750,000 genome-wide variants with respect to Alcohol Use Disorder Identification Test (AUDIT) scores, the ALDH2*2 variant is the only genetic variant influencing the AUDIT score, leading to less alcohol use. In a separate study within this Special Issue, Yi-Chyan Chen et al. measured how the ADH1B*2 and ALDH2*2 genetic variants can impact alcohol metabolism [11]. Sixty males (22–24 years old) were given an alcohol challenge followed by measuring blood ethanol and acetaldehyde levels using gas chromatography and high-performance liquid chromatography. Although the blood ethanol concentration was unchanged by ADH or ALDH2 variants, acetaldehyde accumulation occurred dose-dependently after alcohol consumption in participants with an ALDH2*2 variant. This finding suggests that the ALDH2*2 variant, unlike ADH1B, directly correlated with acetaldehyde accumulation. Taken together, these studies suggest that, regardless of ADH1B genetic polymorphism, the physiological effects of alcohol are directly linked to the inefficient metabolism of acetaldehyde by the ALDH2*2 genetic variant for those of East Asian descent.

With alcohol consumption, the cells lining the upper gastrointestinal tract are directly exposed to alcohol. To further understand the impact, Guidolin et al. used liquid chromatography mass spectroscopy to screen for acetaldehyde-derived DNA adducts in saliva samples collected before and after achieving a 0.11% blood alcohol concentration in 18 non-East Asian human volunteers aged 21–50 years [12]. In this study, a high-resolution accurate mass data-dependent constant neutral loss mass spectroscopy methodology was developed that identified two previously unreported acetaldehyde-induced DNA adducts (*N*^6^-ethyldeoxyadenosine and *N*^4^-ethyldeoxycytidine), in addition to twenty putative acetaldehyde-DNA adducts. In a separate study, Tsai et al. examined in rodents how the ALDH2*2 genetic variant can impact bone loss with dental bacteria (lipopolysaccharides derived from *Polyphyromonas gingivalis*) concomitantly with alcohol [13]. By quantifying the proliferation and mineralization of human osteoblasts in vitro, it was observed that ALDH2 knockdown-transfected osteoblasts grew more slowly (about 5% calcification at day 14), compared to the control-transfected osteoblasts. Furthermore, when wild-type and ALDH2*2 knock-in mice were injected with a purified lipopolysaccharide (LPS) from *P. gingivalis* and given 10% alcohol for 6 weeks, the bone defects in the ALDH2*2 knock-in mice were significantly increased by 30% compared to wild-type ALDH2 mice. This suggests that the ALDH2*2 genetic variant increases the risk of dental bone loss when exposed to alcohol and oral bacteria. In another study, Shimonosono et al. reported that acetaldehyde is associated with alcohol-induced squamous cell carcinoma, suggesting ALDH2-dependent acetaldehyde metabolism prevents cancers associated with alcohol consumption [14]. Acetaldehyde was associated with the post-therapeutic recurrence of alcohol-associated squamous cell carcinoma by mediating apoptosis in normal cells and autophagy in human esophageal squamous cells with increased malignant properties. Together, these articles investigated the direct effects of alcohol metabolism and the generation of acetaldehyde resulting in the formation of DNA and protein adducts within the upper gastrointestinal tract.

Alcohol consumption also impacts the cardiovascular system and prior studies have identified an association between alcohol consumption, genetics, and atrial fibrillation [15,16,17]. In this regard, Hung et al. examined in East Asians the impact of daily alcohol consumption and carrying the ALDH2*2 variant on left atrial electromechanical parameters (including the PR interval, left atrial volume, emptying fraction, and volume index) [18]. Carriers of the ALDH2*2 variant with modest alcohol consumption (~12.3 g daily) demonstrated more impaired left atrial electromechanical parameters and a higher clinical risk CHARGE-AF score (12.7 vs. 5.6) to develop alcohol-associated atrial fibrillation relative to those without. Additionally, the authors reported that circulating 4-hydroxynonenal (4-HNE) was increased in carriers of the ALDH2*2 variant who consume alcohol. Therefore, the authors concluded that 4-HNE accumulation secondary to inefficient alcohol consumption was inversely associated with worsened cardiac function. This may further explain the link between alcohol consumption, inefficient acetaldehyde metabolism, and atrial fibrillation.

### 2.2. Aldehyde Sources in Addition to Alcohol

Aldehydes are also formed intracellularly by lipid peroxidation, including the aldehyde 4-HNE. This is the central theme for 2 original articles and 2 review articles for this issue. The review article by Bilska-Wilkosz et al. summarized the current literature regarding the chemical and biological properties of 4-HNE [19]. Considered generally harmful to humans, 4-HNE also possesses beneficial properties with respect to cancer. For example, higher concentrations of 4-HNE (10–20 micromolar) can promote apoptotic signaling in cancer cells as opposed to lower 4-HNE concentrations (~0.1–5 micromolar) that can trigger cell proliferation and differentiation along with initiating antioxidant defense mechanisms [20,21]. This occurs by triggering aldo-keto reductase family 1-member C1 (AKR1C1) and glutamate cysteine ligase catalytic subunit (GCLC) to reduce ROS-mediated cellular damage [22]. The results indicate that 4-HNE differentially modulates cell death, growth, and differentiation based upon intracellular concentration. Shiau-Mei Chen et al. highlighted how administering 4-HNE and methylglyoxal to mimic hyperglycemia and hyperlipidemia can lead to glucolipotoxicity-induced ROS formation, mitochondrial dysfunction, and β-cell death in MIN6 cells (the cell line derived from mice insulinoma displaying characteristics of pancreatic β-cells) [23]. In this study, the authors reversed β-cell dysfunction by activating ALDH2 with Alda-1 to detoxify 4-HNE-induced mitochondrial dysfunction and β-cell death. In turn, this reversal can limit pancreatic β-cell dysfunction that can progress to type 2 diabetes mellitus. 

Endogenous aldehydes can also trigger pain [24,25]. Martins et al. reported that ALDH2 is an important PKCε substrate in reducing aldehyde-induced hyperalgesia in rodents [26]. The clearance of 4-HNE by ALDH2 mitigated the aldehyde-mediated mechanical hypersensitivity in rodents and mice carrying the ALDH2*2 variant displayed persistent hypersensitivity due to the accumulation of 4-HNE adducts. In addition, Martins et al. demonstrated that 4-HNE caused by oxidative stress from lipid peroxidation in the mitochondria formed protein adducts that are positively correlated with the hypersensitivity in rodents by activating the transient receptor channel ankyrin 1 (TRPA1). Hellenthal et al. summarized the interactions of reactive aldehydes with TRP channels leading to a pro-inflammatory state, suggesting that TRP channels are a potential downstream target of reactive aldehydes [27]. Together, these data highlight the importance of the interactions of aldehydes with TRP channels, which trigger pathologies related to pain and vascular injury [28].

Although much attention focuses on ALDH2 and the ALDH2*2 genetic variant regarding aldehyde metabolism, this enzyme is also only one of 19 enzymes within the aldehyde dehydrogenase family. In this regard, Che-Hong Chen et al. used the global human Genome Aggregation Database (gnomAD) to identify genetic variants for the ALDH family followed by the Polymorphism Phenotyping (PolyPhen) and Sorting Intolerant From Tolerant (SIFT) tools to predict how genetic variants affect structure and function for ALDH enzymes [29]. The key finding was that the ALDH1A gene members (ALDH1A1, ALDH1A2, and ALDH1A3) have the lowest tolerance for loss-of-function mutations as compared to the other ALDH genes, and are much less common in healthy human populations than expected relative to other ALDH enzyme variants.

## 3. ALDH2 STAR Consortium

**Figure 2:**
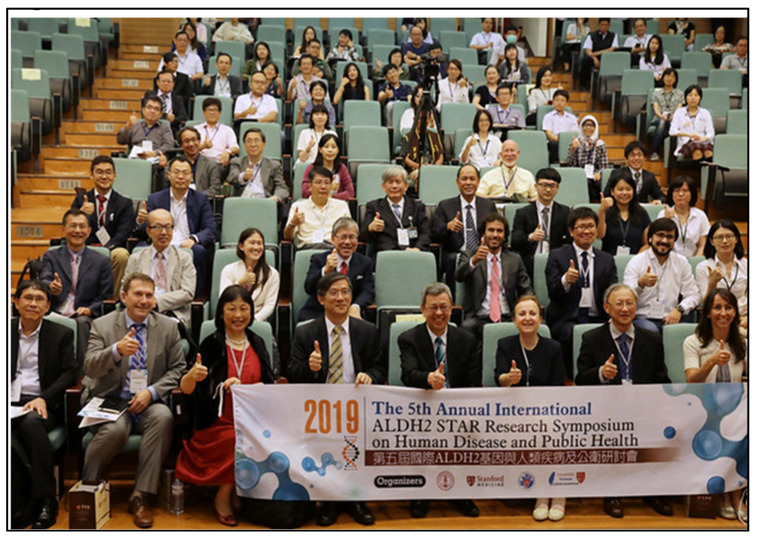
The 5th annual international ALDH2 research symposium on human disease and public health. Group photo on day one of the event.

In order to raise awareness regarding how genetics may influence aldehyde metabolism, the inaugural ALDH2 STAR consortium started with an agreement between Stanford University and Taipei Medical University, Taiwan, in 2015. Spearheaded by Che-Hong Chen and Daria Mochly-Rosen and with grateful support by Yun Yen to host the event at Taipei Medical University, the initial meeting was focused on sharing tools, technology, and scientific expertise between Stanford University and Taiwan as means to gather researchers together who studied topics involving ALDH2. Our consortium continued until the COVID-19 pandemic, with the fifth annual ALDH2 STAR meeting hosted by Kaohsiung Medical University in Kaoshuing, Taiwan, 26–27 September 2019. For this meeting, over 200 people registered for the two-day event representing 6 different countries (Figure 2).

Highlights included opening remarks from Chien-Jen Chen, former Vice-President of Taiwan, and talks from international experts on the topics of precision medicine, cancer, public health, and therapeutic opportunities. 

Starting and continuing this meeting in Taiwan was not a coincidence as Taiwan has the highest prevalence of the inactivating ALDH2 genetic variant in the world (~49%). As such, the ALDH2 STAR meeting has continued to provide a forum for gathering researchers to share scientific results in regard to both aldehyde exposure and aldehyde metabolism. Through the years, the meeting has grown from an initial half-day meeting to a two-day meeting attended by experts in the field, trainees, and public health officials and industry. Together, precision medicine continues to be an important topic to discuss when considering aldehyde production and metabolism. This is because the influence of environmental sources of aldehydes coupled with genetics and lifestyle choices will largely influence the magnitude of the effect that aldehyde exposure may have on human health. This will continue to be a focus of research in the upcoming years. 

## 4. Summary

Taken together, the research and review articles presented here provide an overview of how endogenous aldehyde production, exogenous aldehyde exposure, and aldehyde metabolism impact human health. Research on this topic may open precision medicine pathways and recommendations related to aldehyde exposure, aldehyde metabolism, and the genetics involved in aldehyde metabolism. We hope you enjoy this Special Issue on aldehyde toxicity and metabolism anticipating future ALDH2 STAR consortium meetings.
